# Analysis on the hospitalization expenses structure and correlation degree of lung cancer: evidence from Shanxi Province, China

**DOI:** 10.3389/fpubh.2025.1461789

**Published:** 2026-01-28

**Authors:** Bin Wu, Mingxue Jin, Jing Liu

**Affiliations:** 1 Medical Records Department of Shanxi Province Cancer Hospital, Taiyuan, China; 2 Shanxi Hospital Affiliated to Cancer Hospital, Chinese Academy of Medical Sciences, Taiyuan, China; 3 Cancer Hospital Affiliated to Shanxi Medical University, Taiyuan, China; 4 School of Public Health, Hainan Medical University, Haikou, China

**Keywords:** China, grey correlation analysis, hospitalization expenses, lung cancer, structural change analysis

## Abstract

**Introduction:**

Lung cancer is associated with a heavy economic burden in China. In this study, we analyzed the hospitalization expenses of patients with different types of lung cancer and investigated how the expense structure has changed from 2017 to 2021.

**Methods:**

A total of 17,721 patients were included. All patients with lung cancer discharged from a tertiary hospital in Shanxi, China. Hospitalization expenses were categorized as drug, treatment, medical material, diagnostic, nursing, operating, and other expenses. All expenses were inflated to the price of 2022. New grey correlation degree and structural change analyses were conducted.

**Results:**

Hospitalization expenses increased from 2017 to 2021 at a rate of 27.6%. Among all components, drug expenses were most significantly associated with hospitalization expenses. The expense structure of the hospitalization expenses also changed, with an overall structural variability of 41.74%. The top three contributors to changes were medical material, drug, and treatment expenses. The expense structure differed across lung cancer types.

**Conclusion:**

In summary, the medical expenses of lung cancer patients are increasing. Although the expense structure has changed, there is still plenty of scope for further optimization.

## 1 Introduction

Lung cancer is a significant global health issue, with high morbidity and mortality rates (1, 2). According to the World Cancer Report 2022, lung cancer was the most commonly diagnosed cancer and the leading cause of cancer-related deaths worldwide. In 2022, there were nearly 2.5 million new cases of lung cancer, representing one in eight cancers globally (12.4% of all cancers), and it was responsible for an estimated 1.8 million deaths (18.7% of all cancer deaths) (3). Lung cancer is mainly divided into non-small cell lung cancer and small cell lung cancer, which have significant differences in treatment strategies and clinical prognosis. Expense cell lung cancer is the most common type, and for early patients, surgical resection is the preferred option, which can be supplemented with preoperative neoadjuvant or postoperative adjuvant therapy. Late stage patients rely on systemic therapy such as targeted drugs, immune checkpoint inhibitors, and antibody drug conjugates. Chemotherapy and radiotherapy are the main treatments for small cell lung cancer. Although they are sensitive in the early stages, they are prone to recurrence (27). In addition to its health impact, lung cancer is also associated with a significant economic burden (5), due to its treatment at specialized cancer hospitals. The economic burden of lung cancer has been revealed in various previous studies. For instance, a study conducted in urban China found that the average economic burden from lung cancer was $43,336 per patient, with direct expenses accounting for over 98% of the total expenses (6).

Different types of lung cancer have varied treatment options, and the prognosis is generally poor (5). Although significant progress has been made in the treatment of early non-small cell lung cancer with improved prognosis, overall, the prognosis of lung cancer is still poor due to the fact that most patients are diagnosed in advanced stages. Moreover, China has the highest lung cancer burden globally, with approximately 37% of global cases and 39% of deaths attributable to lung cancer (7). The Chinese government has implemented a series of policies and activities to reduce the economic burden of lung and other cancers by optimizing hospitalization expense structures, so as to improve healthcare system efficiency and provide equitable access to healthcare services (8). In particular, the Centralized Volume-Based Procurement (CVBP) policy, aimed at ensuring public access to higher-quality medicines at lower prices (9), has undergone continuous intensification and expansion. For instance, following our study period, Shanxi Province launched a comprehensive initiative to extend CVBP drug coverage to primary care institutions, private hospitals, and retail pharmacies (10). Such post-2021 policy evolutions are fundamentally reshaping the pharmaceutical market and cost structures, establishing a distinct context for later periods (9).

Previous studies have examined the economic burden of lung cancer and the influencing factors associated with the expenses (11–13). In addition, some have investigated how the expense structure changed in the context of China’s healthcare reform (14–16). However, these studies did not specifically focus on the expense structure of different types of lung cancer, which is a critical issue in healthcare management. Understanding the expense structure of different types of lung cancer hospitalization expenses is imperative for healthcare policymakers to optimize healthcare resource allocation and improve healthcare system efficiency. This gap in the literature emphasizes the necessity for more research in the area of the expense structure of different types of lung cancer.

In response to this research gap, the current study aims to analyze the hospitalization expenses of lung cancer patients from 2017 to 2021 by means of new grey correlation degree and structural change analyses. The study aims to provide evidence for decision-making regarding hospital management and adjustment of the structure of medical services. Specifically, the study seeks to examine the impact of medical service price reform on the expense structure of lung cancer hospitalization expenses in Shanxi, a province in North China, which has made a breakthrough in controlling hospitalization expenses at medical institutions by abolition of drug mark-ups.

In summary, lung cancer is a significant global health issue associated with high morbidity and mortality rates and a heavy economic burden. The Chinese government has implemented various policies and activities to reduce the economic burden of lung cancer and improve healthcare system efficiency. While previous studies have examined the economic burden of lung cancer and healthcare system reform in China, there is a need for more research on the hospitalization expense structure of different types of lung cancer. The current study aims to fill this research gap by analyzing the hospitalization expense structure of different types of lung cancer in Shanxi Province, China.

## 2 Materials and methods

### 2.1 Data collection

The medical records of patients with a first diagnosis of lung cancer, who were discharged from the hospital from 2017 to 2021, were collected from the hospital information management system of Shanxi Province Cancer Hospital. The diagnosis of lung cancer is based on the code C34 in the Tenth Revision of the International Classification of Diseases. The histopathological diagnosis of all cases was confirmed by senior doctors in the hospital’s pathology department, and the diagnostic criteria were consistent with the clinical practice guidelines at that time (including the WHO classification principles released in 2021) to ensure consistency in diagnosis. The collected data included patients’ demographics (age and sex), clinical information (length of stay and treatment schedules), and hospitalization expenses. The hospitalization expenses were categorized into different components, including drug expenses, diagnostic expenses, treatment expenses, nursing expenses, surgical expenses, medical material expenses, and other expenses. The expenses were adjusted for inflation based on the consumer price index (CPI) of Shanxi Province in 2022. Data quality was checked and evaluated according to the criteria of Chinese Cancer Registration and IARC/IACR (17).

### 2.2 Study participants

**Figure 1:**
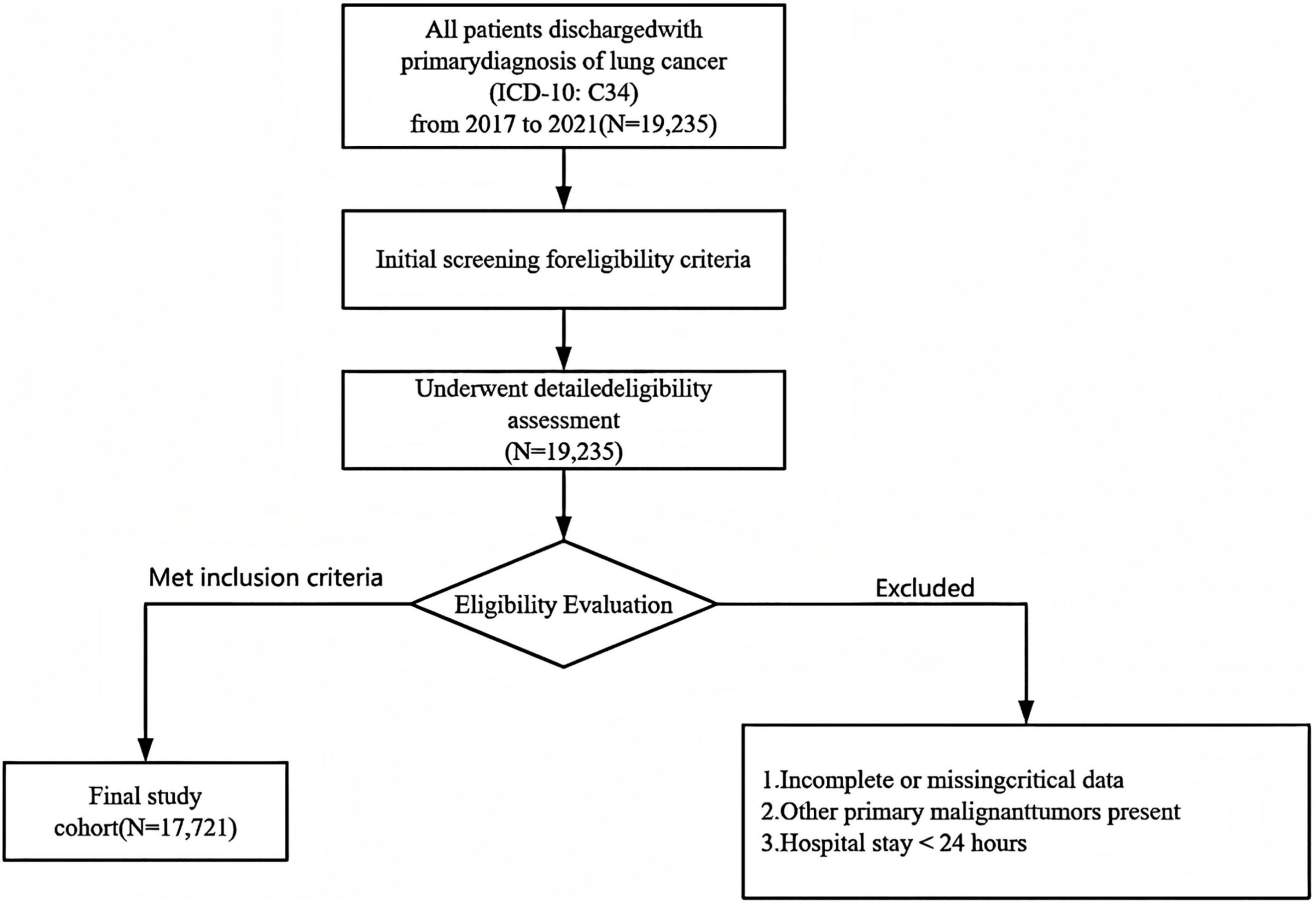
Flowchart of study patient selection.

The study included 17,721 patients who were discharged from the hospital between 2017 and 2021 with a first diagnosis of lung cancer, ICD-10 code C34 for Lung Tumors. The inclusion criteria are as follows: (1) patients primarily diagnosed with lung cancer (ICD-10: C34); (2) First hospitalization due to lung cancer; (3) Medical records and expense data are complete; and (4) This study only included primary lung cancer (ICD-10 code C34). Excluded lung metastatic tumors, mesothelioma, and tumors with unknown primary sites. The exclusion criteria are as follows: (1) patients with incomplete or missing data; (2) Patients with other primary cancers; and (3) Patients who have been hospitalized for less than 24 h (Figure 1).

### 2.3 Variables

The following demographic and clinical variables were analysed in this study: patients’ demographics (age and sex), clinical information (length of stay and treatment regimens), and hospitalization expenses. The hospitalization expenses were categorized into different components, including drug expenses, diagnostic expenses, treatment expenses, nursing expenses, surgical expenses, medical material expenses, and other expenses. To ensure clarity, each expense category is explicitly defined as follows; Drug expenses: Costs of all pharmaceuticals, including Western medicines and Chinese patent medicines. Treatment expenses: Costs of non-surgical therapeutic procedures, including radiotherapy, chemotherapy, infusion therapy, and interventional therapy; Medical material expenses: Costs of consumables and implants, such as syringes, sutures, surgical staplers, and stents; Diagnostic expenses: Costs of examinations and tests used for diagnostic purposes, including imaging (e.g., CT, MRI), laboratory tests, and pathological examinations; Nursing expenses: Costs associated with levels of nursing care; Surgical expenses: Costs directly related to surgical procedures, including surgeon fees and anesthesia; Other expenses: Costs not covered by the above categories. The primary outcomes were the total hospitalization expenses and their annual growth rate. In addition, complications that occur during treatment, such as infections and costs incurred from intensive care, are classified as infection treatment costs or intensive care costs based on their nature. Special treatments for metastatic lesions, such as cranial radiation therapy and radiofrequency ablation of liver metastases, are included in the treatment fee, including treatment operation fees, equipment usage fees, and related consumables costs. The cost of re biopsy after disease progression is included in the diagnosis fee, and the cost of drugs generated by adjusting the new treatment plan due to subsequent gene mutation analysis is included in the drug fee (Table 1).

**Table 1:** Expense components.

	Category	Definition and components
Hospitalization expenses	Drug expenses	Costs of all pharmaceuticals, including Western medicines and Chinese patent medicines. New medication after genetic mutation analysis
	Treatment expenses	Costs of non-surgical therapeutic procedures, including radiotherapy, chemotherapy, infusion therapy, and interventional therapy.
	Medical material expenses	Costs of consumables and implants, such as syringes, sutures, surgical staplers, and stents.
	Diagnostic expenses	Costs of examinations and tests used for diagnostic purposes, including imaging (e.g., CT, MRI), laboratory tests, and pathological examinations. Re-biopsy after progression
	Nursing expenses	Costs associated with levels of nursing care.
	Surgical expenses	Costs directly related to surgical procedures, including surgeon fees and anesthesia.
	Other expenses	Costs not covered by the above categories.
Additional notes	Complications related expenses	Infection treatment costs and intensive care costs are classified into corresponding categories based on their nature
	Special treatments for metastatic lesions	Cranial radiation therapy, liver metastasis radiofrequency ablation, etc., are included in the treatment costs (including operating fees, equipment usage fees, and related consumables)

### 2.4 Statistical analysis

Descriptive analyses were performed to summarize the yearly hospitalization expenses data. Because it is suitable for measuring the consistency of development trends over time in small sample time series. Exploring the correlation between factors affecting hospitalization costs and hospitalization costs using new grey relational analysis (18). The structural change analysis method was applied to analyze the longitudinal data of hospitalization expenses. This method can reflect the dynamic change trend of various expenses and the overall characteristics of the structural changes comprehensively (19, 20). The principle is to measure the geometric similarity between a reference sequence (in this study, the annual total hospitalization expenses) and several comparison sequences (i.e., the annual costs of each expense component, such as drug costs, material costs, etc.). The higher the correlation, the more consistent the dynamic development trend of the comparison sequence is with the reference sequence, indicating a stronger correlation between the two. The indicators of this method include Value of Structure Variation (VSV), Degree of Structure Variation (DSV), and Structural Contribution rate. A positive VSV indicates that the item composition ratio is increasing, while a negative VSV indicates that the item composition ratio is decreasing. As DSV=∑∣VSV∣ , the larger the fluctuation value of structural fluctuation is, the greater the degree of structural fluctuation of this period will be; the structural change contribution rate is calculated by means of the formula below (15):


Contribution rate=∣Xi1−Xi0∣/DSV×100%

Statistical analyses and graphical representations were conducted by Excel 2010 and SPSS version 26.0 software (SPSS, Chicago, IL, United States). *p* < 0.05 were considered statistically significant.

## 3 Results

### 3.1 Patients’ characteristics

A total of 17,721 patients were included, including 3,136 in 2017, 3,521 in 2018, 3,995 in 2019, 3,547 in 2020, and 3,522 in 2021. The patient characteristics are shown in Table 2. The mean (SD) age of the patients was 64.78 (9.31) years, and 28.2% were female. On average, length of stay of patients were 22.55 days (Table 2).

**Table 2:** Characteristics of patients.

Characteristic	Sample included in analysis (*N* = 17,721)
Age (year), mean ± SD	64.78 (9.31)
Female, *n* (%)	28.2%
Length of stay (day), mean ± SD	22.55 (17.01)
Year of discharge, *n* (%)	
2017	3,136 (17.7)
2018	3,521 (19.9)
2019	3,995 (22.5)
2020	3,547 (20.0)
2021	3,522 (19.9)

### 3.2 Descriptive analysis

The average hospitalization expenses in 2017 and 2021 are summarized in Table 3. The expenses increased from ¥33,472.95 in 2017 to ¥42,703.41 in 2021, with an absolute increase of ¥9,239 and an increase rate of 27.6%. Regarding patients with different types of lung cancer, the highest growth rate was observed for those with adenocarcinoma (26.6%), followed by squamous cell carcinoma (4.72%). In contrast, the average hospitalization expenses for patients with small-cell carcinoma decreased by 12.65% (Table 3).

**Table 3:** Average hospitalization expenses for patients with lung cancer in 2017 and 2021.

Type of lung cancer	Average hospitalization expense, Chinese Yuan ¥					Increment, ¥	Growth rate, %
	2017	2018	2019	2020	2021		
Overall	33472.95	34806.36	38131.77	38336.73	42703.41	9230.46	27.6
Small-cell carcinoma	26277.49	23396.63	24431.79	22330.95	22953.39	−3324.10	−12.65
Squamous cell carcinoma	39222.22	37508.23	42382.47	39677.64	41073.40	1851.18	4.72
Adenocarcinoma	41292.59	42249.17	47257.85	46676.04	52,279	10986.41	26.61
Large-cell carcinoma	63545.36	55079.1	55528.66	47372.79	62227.74	−1317.62	−2.07
Other types	29178.70	31719.51	26371.33	27682.27	31614.35	2435.65	8.35

**Figure 2:**
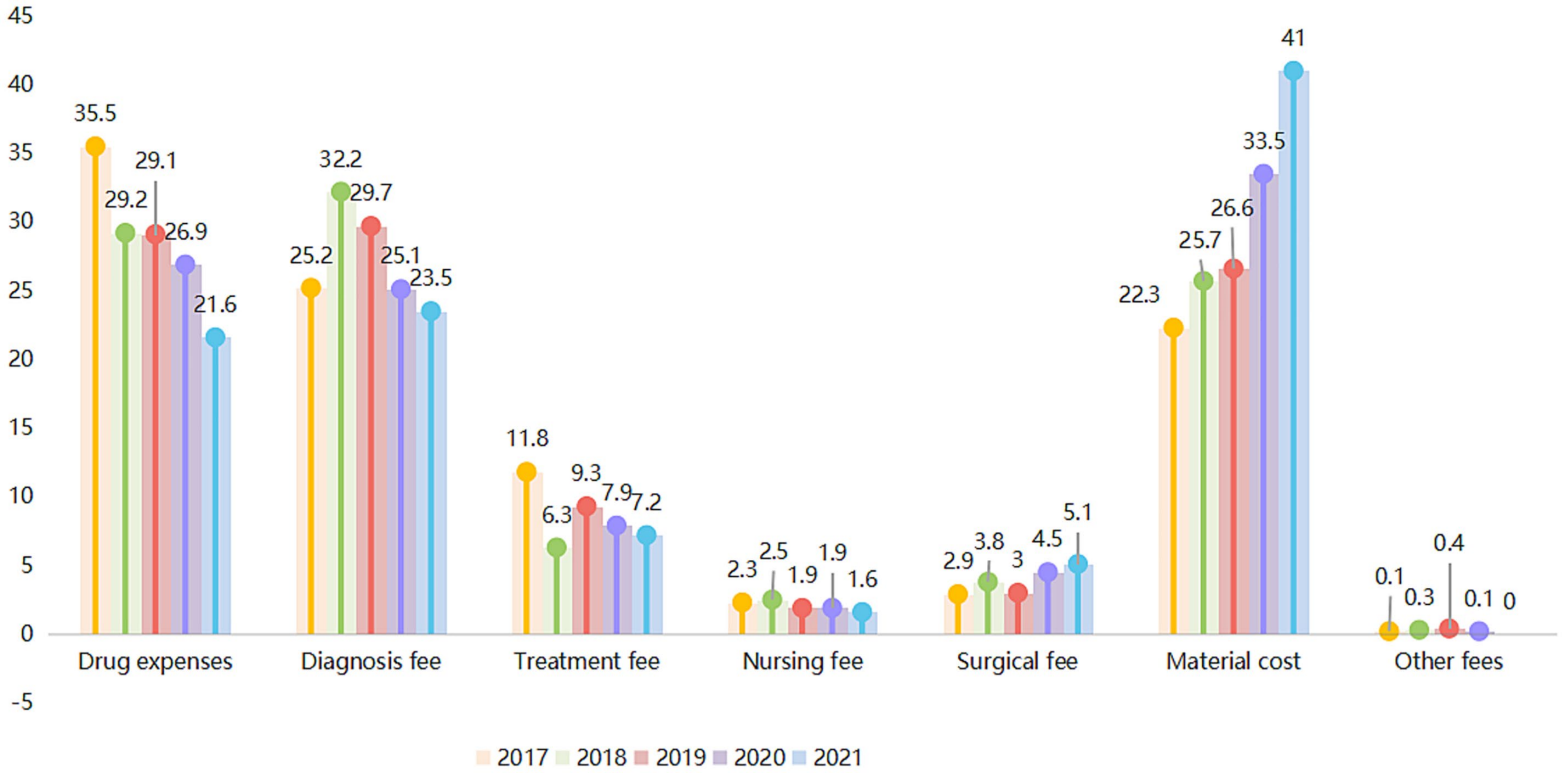
Expense structure of average hospitalization expenses for patients with lung cancer from 2017 to 2021.

The structure of the expense components in the average hospitalization expenses of lung cancer patients has changed significantly from 2017 to 2021, as indicated by the proportion of each expense component shown in Figure 2. The proportion of medical material expenses has increased yearly, while the proportion of drug expenses has decreased yearly. Specifically, compared to 2017, the medical material expenses in 2021 increased by 18.7% (41% in 2021 and 22.3% in 2017), while the proportion of drug expenses decreased by 13.9% (21.6% in 2021 and 35.5% in 2017), the proportion of diagnostic expenses decreased by 1.7% (23.5% in 2021 and 25.2% in 2017), and the proportion of treatment costs expenses decreased by 4.6% (7.2% in 2021 and 11.8% in 2017).

### 3.3 New grey correlation analysis

**Figure 3:**
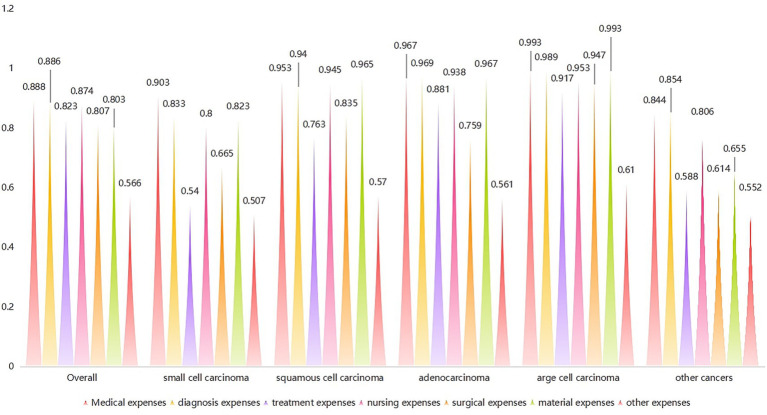
Degree of association of type of lung cancer and expense types.

The new grey correlation analysis of the 2017–2021 data suggested that drug expenses were most significantly associated with the overall lung cancer (degree of association: 0.888) (Figure 2). The same result was also observed for patients with small-cell lung carcinoma (degree of association: 0.903) and those with large-cell carcinoma (degree of association: 0.993). For patients with squamous cell carcinoma, treatment expenses were the component with the highest correlation (degree of association: 0.965), while for patients with adenocarcinoma, diagnostic expenses were the most significant component (degree of association: 0.969) (Figure 3).

### 3.4 Analysis of structural change

From 2017 to 2021, the overall structural variability of the average hospitalization expenses was 41.74%. For different types of lung cancer, the structural changes observed in descending order were 38.09% in adenocarcinoma, 37.50% in small-cell carcinoma, 20.78% in squamous cell carcinoma, and 20.67% in large-cell carcinoma (Table 4).

**Table 4:** Structural change in average hospitalization expenses for patients with lung cancer from 2017 to 2021.

Expense Component	Structural change value							Structural variability
	Drug expenses	Treatment expenses	Medical material expenses	Diagnostic expenses	Nursing expenses	Operating expenses	Other expenses	
Overall	−13.90	−4.59	18.70	−1.71	−0.63	2.17	−0.05	41.74
Small-cell carcinoma	−5.84	−12.78	7.35	10.55	−0.02	0.85	−0.11	37.50
Squamous cell carcinoma	−2.78	−7.41	2.92	5.16	−0.16	2.31	−0.04	20.78
Adenocarcinoma	−12.31	−1.23	17.16	−4.88	−0.60	1.88	−0.03	38.09
Large-cell carcinoma	−5.27	−4.59	9.78	0.36	−0.47	0.20	−0.01	20.67
Other types	−15.34	−6.17	20.28	−0.85	−0.58	2.74	−0.08	46.04

In addition, drug, treatment, diagnostic and nursing expenses all showed negative changes among all lung cancer patients, while medical material and operating expenses showed positive changes (Table 4). Regarding small-cell and squamous cell carcinoma, medical material, diagnostic, and operating expenses showed positive changes, while the other expenses showed negative changes; the largest change was observed in medical material expenses (−12.78 for small-cell carcinoma and −7.41 for squamous cell carcinoma). Regarding adenocarcinoma, positive changes were observed in materials and operating expenses, and the largest change was in medical material expenses (17.16), followed by drug expenses (−12.31). Regarding large-cell carcinoma, medical material, diagnostic, and operating expenses showed positive changes, and the largest change was in material expenses (9.78) (Table 4).

### 3.5 Analysis of the contribution rate of structural change

**Figure 4:**
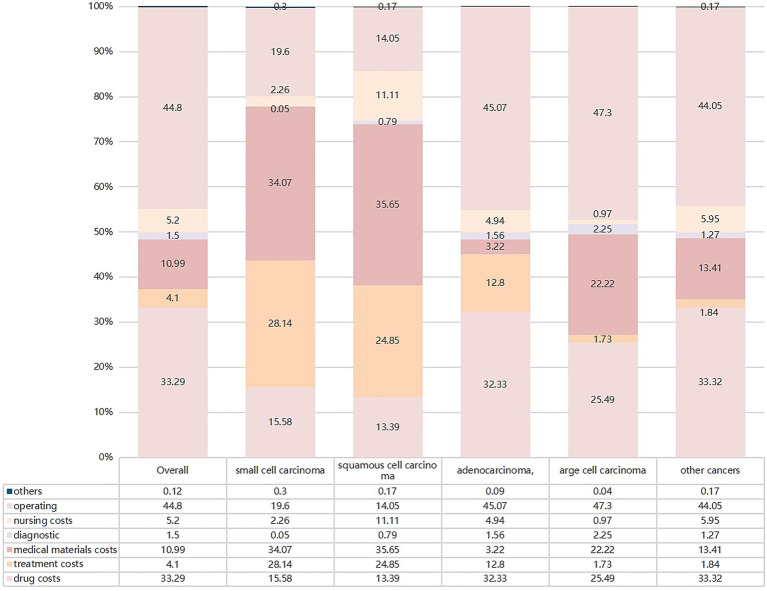
Contribution rate of structural change.

From 2017 to 2021, the top three contributors to changes in the structure of overall hospitalization expenses were material (44.8%), drug (33.29%), and treatment (10.99%) expenses (Figure 4). Treatment expenses were the largest contributors to small-cell carcinoma and squamous cell carcinoma expenses (34.07 and 35.65%, respectively). Regarding adenocarcinoma and large-cell carcinoma, the top contributor was medical material expenses (45.07 and 47.30%, respectively) (Figure 4).

## 4 Discussion

The descriptive analysis revealed that total hospitalization expenses increased by 27.6% from 2017 to 2021, while the proportion of these expenses attributed to drugs decreased significantly. Such findings are consistent with the global and domestic increase in lung cancer prevalence (22–24) and results reported by Guo et al. (25) and Chen et al. (26). The rising drug expenses are associated with the long treatment cycles required for lung cancer, complex treatment protocols and long hospital stays, resulting in serious consumption of healthcare resources (30). The rising expenses of pharmaceuticals also contribute to this drug expense increase. Increasing hospitalization expenses could worsen the heavy economic burden of lung cancer patients. Therefore, there is an urgent need to control these expenses.

Our further analyses of expense structure showed that medical material expenses are increasing, while the proportion of drug expenses is decreasing. The results supported by the analysis of structural changes, indicated the negative changes in drug expense. Overall, it is clear that drug expenses have been effectively controlled between 2017 and 2021, potentially due to healthcare reform measures such as drug mark-up abolition and the implementation of Diagnosis Related Group (DRG) payment in Shanxi Province. The medical material expenses have shown a positive change, suggesting an increasing trend of this component in overall hospitalization expenses. The large demand for consumables for lung cancer patients and consumable price increases may explain this change. Patients’ preference for consumables may also contribute. Patients have become more aware of their health and are more willing to pay for expensive consumables. This may also reflect the increased adoption of minimally invasive surgeries and high-value implants, alongside potential patient preferences for advanced materials. Nevertheless, it should be noted that in 2021, medical material expenses are the main contributing factor to overall hospitalization expenses, accounting for 41.0%. This finding may provide insights for the next step of healthcare reform and suggest a need to focus on consumables, in order to control the total expenses of lung cancer patients (7).

Notably, there were remarkable differences in hospitalization expense structure changes among patients with different types of lung cancer. For instance, patients with adenocarcinoma exhibited the highest growth rate in hospitalization expenses (26.6%), which may be attributed to the widespread use of targeted therapies and immunotherapies, along with more frequent genetic testing and personalized treatment regimens. This observation aligns with the global shift towards novel, high-cost therapeutic agents for advanced non-small cell lung cancer (28). Although targeted drugs and immunotherapies are likely a significant driver behind the rising drug expenses, it must be acknowledged that our dataset did not permit systematic differentiation of specific treatment modalities. Consequently, the costs of these innovative drugs are aggregated within the broad category of “drug expenses,” and their individual impact warrants further investigation in future studies.

Simultaneously, the marked increase in the proportion of medical material expenses (rising from 22.3% in 2017 to 41.0% in 2021) deserves heightened attention. As defined in our study, this category encompasses all high-value consumables and implants used in surgical and interventional procedures, such as surgical staplers and stents. Therefore, this upward trend not only reflects the growing adoption of minimally invasive techniques but may also be associated with rising costs of the materials themselves and patient preference for higher-quality options. It is important to note that the professional service value of the surgical procedures is captured separately under “surgical expenses,” with both components together constituting the total cost associated with operative care.

In contrast, the decrease in expenses for small-cell carcinoma (−12.65%) might be related to shorter hospital stays and the shift towards outpatient chemotherapy and palliative care. These differences highlight the necessity of tailoring cost-control policies and clinical pathways according to the specific biological behavior and treatment patterns of each lung cancer subtype. The average hospitalization expense structure changes for patients with lung cancer are enormous, as are changes in the contribution of different expense components. Given these differences, it is suggested that hospitals consider the treatment characteristics of different types of lung cancer and optimize clinical treatment pathways. With these measures in effect simultaneously, it is hoped that high-quality healthcare services can be provided with controlled expenses.

In terms of changes in expense structure, negative changes were observed in diagnostic, treatment, and nursing expenses. Such findings are unsurprising. After the “Notice on Promoting the Reform of Medical Service Price Reform” issued in 2016, Shanxi Province initiated a series of reforms, such as adjusting the price of medical services, including diagnosis, treatment, surgery, rehabilitation, and nursing, to better reflect the value of medical professional labor. Nevertheless, the changes were relatively small. And the price adjustment of medical services has yet to optimize the structure of hospitalization expenses or reflect the labor value of medical professionals effectively. Therefore, it is recommended that a dynamic price adjustment mechanism based on changes in the expense and income structure should be established by the joint efforts from National Development and Reform Commission, the Price Bureau, the Medical Insurance Bureau, the Health Commission, and hospitals in China, to resolve the price comparison relationship of medical services gradually and reflect the labor value of medical professionals. Hospitals may also consider thorough reforms in standardizing diagnosis and treatment behaviors and controlling expenses, focusing on providing high-quality healthcare services.

Interpreting these findings requires an understanding of the broader context of China’s ongoing healthcare system reforms. The Chinese healthcare system, predominantly public hospital-based, is undergoing a profound transformation in payment methodologies, with the nationwide rollout of the Diagnosis-Related Group (DRG)-based payment system at its core. This reform aims to incentivize hospitals to proactively control costs and improve efficiency through a prospective payment model. Furthermore, following the 2016 national guideline “Notice on Promoting the Reform of Medical Service Prices,” provinces including Shanxi the site of this study have systematically adjusted medical service prices. A key objective is to dismantle the legacy mechanism of relying on drug markups for hospital revenue and to better reflect the value of healthcare professionals’ knowledge and labor. The observed decline in the proportion of drug expenses and the concurrent rise in material expenses captured in this study serve as a vivid snapshot of the initial effects of these intertwined reforms, which may involve cost-shifting between categories (29).

Based on these insights, we believe there remains considerable scope for optimizing the hospitalization expense structure for lung cancer. The “thorough reforms” we suggest should encompass two complementary levels. First, at the macro-policy level, a dynamic medical service price adjustment mechanism should be established, requiring coordinated efforts from agencies such as the Healthcare Security Administration, the National Health Commission, and the National Development and Reform Commission. This mechanism should enable regular assessment of changes in cost structures and scientifically adjust the prices of diagnostic, surgical, nursing, and other services to ensure they adequately reflect the professional value of medical labor. Second, at the micro-implementation level, healthcare institutions should develop and strictly implement refined clinical pathways tailored to the distinct biological behaviors and treatment patterns of different pathological subtypes (e.g., adenocarcinoma vs. squamous cell carcinoma), in accordance with national clinical practice guidelines for lung cancer. Standardizing diagnosis and treatment behaviors in this way can effectively reduce unnecessary examinations and consumable use, thereby achieving cost control without compromising the quality of care. Similar trends in the drivers of lung cancer hospitalization expenses have been observed in other regions of China (4).

The structural analysis of the average hospitalization expenses for patients with different types of lung cancer focused on the internal composition of the expense, and the correlation analysis focused on the correlation between the expense and its various expense components. The research findings could inform hospital reform policy making in China. However, the findings must be further verified due to the lack of analysis of other factors.

## 5 Conclusion

The longitudinal data analyzed in this study revealed that the hospitalization expenses of patients with lung cancer are increasing, and the expense structure has changed since the China healthcare reform. However, there is still plenty of scope for the expense structure to be further optimized to better reflect the labor value of medical professionals. The overall expense structure and changes in this expense structure differed among patients with different types of lung cancer. Thus, we recommend that hospitals in China further optimize clinical pathways to provide high-quality healthcare services to patients.

It should be noted that the data for this analysis cover the period from 2017 to 2021, which largely precedes the full-scale, post-2021 intensification of the CVBP policy, including significant provincial expansions such as the “Access to Primary Care Institutions, Private Hospitals, and Retail Pharmacies” initiative in Shanxi (10). Consequently, the findings and cost structure trends described herein are most indicative of the phase preceding this latest wave of policy deepening. Evaluating the impact of these recent, comprehensive CVBP implementations on lung cancer hospitalization economics presents a critical avenue for future research (9).

At the same time, this study also compared the absolute value of average hospitalization expense for lung cancer patients between 2017 and 2021, as shown in Table 5. From 2017 to 2021, the absolute value of average hospitalization costs increased by 27.6%. However, its internal composition has undergone drastic changes: although drug costs have absolutely decreased by 22.3%, consumables costs have experienced rapid growth, with an absolute increase of up to 134.6%. This indicates that the focus of medical resource consumption has significantly shifted from drugs to medical consumables. The high average cost growth rate of 26.6% in lung adenocarcinoma patients, as well as the high correlation between diagnosis and drug costs in grey relational analysis, indicate that the cost structure of adenocarcinoma is typically characterized by a pattern of high diagnosis and high drug costs. The cost pattern of squamous cell carcinoma of the lung depends on the significant decrease in total hospitalization costs (−36.6%) observed in patients with small cell lung cancer undergoing radiotherapy and chemotherapy. Small cell lung cancer has the characteristics of high malignancy and rapid progression, and treatment is based on systemic chemotherapy and radiotherapy. The decrease in its cost may reflect two changes in clinical practice: first, the increasing standardization and normalization of treatment; second, due to considerations of quality of life and medical efficiency, chemotherapy and supportive treatment are increasingly being completed in day wards or outpatient clinics, resulting in a decrease in the duration and intensity of single hospitalizations. This means that the medical economic burden of small cell lung cancer may be shifting from the inpatient system to the outpatient system.

**Table 5:** Absolute comparison of average hospitalization costs for lung cancer patients in 2017 and 2021 (RMB, yuan).

Expense category	Average cost per transaction in 2017	Average cost per transaction in 2021	Absolute change value	Absolute rate of change
Total cost	33472.95	42703.41	9230.46	+27.6%
Medication cost	10169.77	8812.27	−1357.5	−13.35%
Consumables fee	6392.50	16675.2	10,282.7	+160.84%
Treatment fee	3373.68	3584.43	210.75	+6.25%
Diagnosis fee	7279.13	9558.01	2,278.89	+31.30%
Surgical fee	831.26	2061.66	1,230.4	+148%
Nursing fee	645.59	662	16.41	+2.54%
Other fees	26.88	18.02	−8.86	−32.96%

The findings of this study are based on the background of China’s healthcare system, which is characterized by a system dominated by public hospitals and an ongoing reform of grouping fees based on disease diagnosis. The medical insurance policies and treatment practices may vary in different countries, so caution should be exercised when applying the conclusions of this study to other environments. Future research should conduct cross-border comparisons to provide more universal cost management insights. One limitation of this study is that the data used is up to 2021. We were unable to systematically collect and differentiate specific treatment modalities, such as surgery, chemotherapy, radiotherapy, targeted therapy, and immunotherapy. Although more recent data cannot be obtained, the period from 2017 to 2021 covers key healthcare reform policies such as zero markup on drugs and DRG payment reform in China, providing valuable baseline data for evaluating the initial impact of these policies on the hospitalization cost structure of lung cancer and laying the foundation for future tracking research.

## Data Availability

The original contributions presented in the study are included in the article, further inquiries can be directed to the corresponding author.
